# Tetra-μ-acetato-κ^8^ *O*:*O*′-bis­[(2-amino-3,5-dichloro­pyridine-κ*N* ^1^)copper(II)](*Cu*—*Cu*)

**DOI:** 10.1107/S1600536811015662

**Published:** 2011-05-07

**Authors:** Hui-Chang Chang, Jacqueline M. Cole, Tze-Chia Lin, Paul G. Waddell

**Affiliations:** a Cavendish Laboratory, University of Cambridge, J. J. Thomson Avenue, Cambridge CB3 0HE, England

## Abstract

The title binuclear Cu(II) complex, [Cu_2_(CH_3_CO_2_)_4_(C_5_H_4_Cl_2_N_2_)_2_], is disposed about a crystallographic inversion center, located at the mid-point of the Cu—Cu connecting line. The Cu⋯Cu distance is 2.6600 (6) Å and each metal atom exhibits a Jahn–Teller-distorted octa­hedral geometry.

## Related literature

For the structures of polymorphic tetra­kis­(*μ*-acetato-*O*:*O*′)bis­[(pyridine-*N*)copper(II)], see: Barclay & Kennard (1961 ▶); Hanic *et al.* (1964 ▶); Uekusa *et al.* (1989 ▶). 
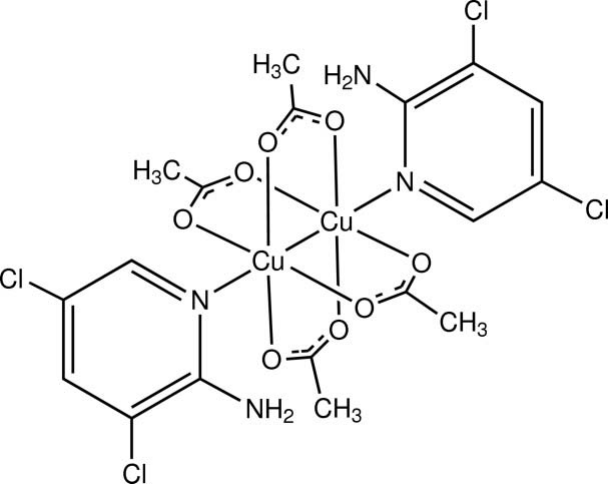


## Experimental

### 

#### Crystal data

[Cu_2_(C_2_H_3_O_2_)_4_(C_5_H_4_Cl_2_N_2_)_2_]
*M* *_r_* = 689.26
Monoclinic, 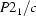

*a* = 8.2857 (17) Å
*b* = 17.010 (3) Å
*c* = 9.3159 (19) Å
β = 103.07 (3)°
*V* = 1279.0 (4) Å^3^
*Z* = 2
Mo *K*α radiation
μ = 2.13 mm^−1^
*T* = 150 K
0.44 × 0.37 × 0.17 mm

#### Data collection

Rigaku Saturn724+ diffractometer
Absorption correction: multi-scan (*ABSCOR*; Higashi, 1995 ▶) *T*_min_ = 0.407, *T*_max_ = 0.696
18645 measured reflections
3028 independent reflections
2996 reflections with *I* > 2σ(*I*)
*R*_int_ = 0.032

#### Refinement

*R*[*F* ^2^ > 2σ(*F* ^2^)] = 0.029
*wR*(*F* ^2^) = 0.077
*S* = 1.10
3028 reflections
166 parameters
H-atom parameters constrained
Δρ_max_ = 0.51 e Å^−3^
Δρ_min_ = −0.35 e Å^−3^

### 

Data collection: *CrystalClear* (Rigaku/MSC, 2008 ▶); cell refinement: *CrystalClear*; data reduction: *CrystalClear*; program(s) used to solve structure: *SHELXS97* (Sheldrick, 2008 ▶); program(s) used to refine structure: *SHELXL97* (Sheldrick, 2008 ▶); molecular graphics: *SHELXTL* (Sheldrick, 2008 ▶); software used to prepare material for publication: *WinGX* (Farrugia, 1999 ▶).

## Supplementary Material

Crystal structure: contains datablocks global, I. DOI: 10.1107/S1600536811015662/bh2343sup1.cif

Structure factors: contains datablocks I. DOI: 10.1107/S1600536811015662/bh2343Isup2.hkl

Additional supplementary materials:  crystallographic information; 3D view; checkCIF report

## Figures and Tables

**Fig. 1.**
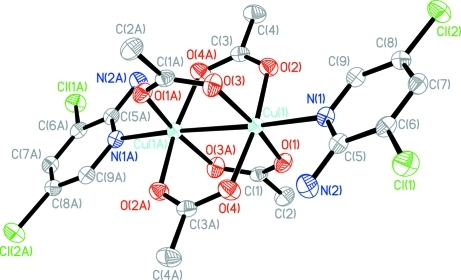
The structure of the title compound with displacement ellipsoids drawn at the 50% probability level; hydrogen atoms omitted for clarity. Atoms labeled with flag A are generated by symmetry 1-x, 1-y, 1-z.

## supplementary crystallographic information

### Comment

The title compound is binuclear and disposed about a crystallographic centre of symmetry with a Cu—Cu distance of 2.6600 (8) Å. It has a similar geometry to that observed in the two known polymorphs of monopyridinecopper(II) acetate (Barclay & Kennard, 1961; Hanic *et al.*, 1964; Uekusa *et al.*, 1989). However, the Cu—N bond distance in the title compound is *ca*. 0.05 Å longer than that observed in the orthorhombic polymorph and *ca*. 0.08 Å longer than that in the monoclinic polymorph.

### Experimental

A suspension of (3,5-dichloro-2-pyridylimino)-*o*-cresol copper (II) (1 mg, 0.0016 mmol) in ethanol (*ca*. 3 ml) was heated to *ca*. 323 K until fully dissolved. The solution was then allowed to cool to room temperature. Crystals suitable for single-crystal X-ray crystallography were grown *via* slow evaporation of methanol over seven days.

### Refinement

All H atoms were placed in idealized positions and refined as riding to their parent atoms, with bond lengths fixed to C—H = 0.93 (aromatic CH), 0.96 (methyl CH*_3_*) or 0.86 Å (amine NH_2_). Isotropic displacement parameters were calculated as *U*_iso_(H) = 1.5 *U*_eq_(carrier atom) for methyl groups and *U*_iso_(H) = 1.2 *U*_eq_(carrier atom) otherwise.

### Crystal data

**Table:**

[Cu_2_(C_2_H_3_O_2_)_4_(C_5_H_4_Cl_2_N_2_)_2_]	*F*(000) = 692
*M**_r_* = 689.26	*D*_x_ = 1.79 Mg m^−^^3^
Monoclinic, *P*2_1_/*c*	Mo *K*α radiation, λ = 0.71073 Å
Hall symbol: -P 2ybc	Cell parameters from 6896 reflections
*a* = 8.2857 (17) Å	θ = 4.8–36.7°
*b* = 17.010 (3) Å	µ = 2.13 mm^−^^1^
*c* = 9.3159 (19) Å	*T* = 150 K
β = 103.07 (3)°	Prism, blue
*V* = 1279.0 (4) Å^3^	0.44 × 0.37 × 0.17 mm
*Z* = 2	

### Data collection

**Table:**

Rigaku Saturn724+ diffractometer	3028 independent reflections
Radiation source: fine-focus sealed tube	2996 reflections with *I* > 2σ(*I*)
graphite	*R*_int_ = 0.032
Detector resolution: 28.5714 pixels mm^-1^	θ_max_ = 28.3°, θ_min_ = 4.8°
ω scans	*h* = −11→11
Absorption correction: multi-scan (*ABSCOR*; Higashi, 1995)	*k* = −22→20
*T*_min_ = 0.407, *T*_max_ = 0.696	*l* = −12→12
18645 measured reflections	

### Refinement

**Table:**

Refinement on *F*^2^	Primary atom site location: structure-invariant direct methods
Least-squares matrix: full	Secondary atom site location: difference Fourier map
*R*[*F*^2^ > 2σ(*F*^2^)] = 0.029	Hydrogen site location: inferred from neighbouring sites
*wR*(*F*^2^) = 0.077	H-atom parameters constrained
*S* = 1.10	*w* = 1/[σ^2^(*F*_o_^2^) + (0.0389*P*)^2^ + 0.9376*P*] where *P* = (*F*_o_^2^ + 2*F*_c_^2^)/3
3028 reflections	(Δ/σ)_max_ = 0.001
166 parameters	Δρ_max_ = 0.51 e Å^−^^3^
0 restraints	Δρ_min_ = −0.35 e Å^−^^3^
0 constraints	

### Fractional atomic coordinates and isotropic or equivalent isotropic displacement parameters (Å^2^)

**Table:**

	*x*	*y*	*z*	*U*_iso_*/*U*_eq_	
Cu1	0.42778 (2)	0.430189 (12)	0.47068 (2)	0.01965 (8)	
Cl1	0.39574 (7)	0.08106 (3)	0.48552 (6)	0.03401 (12)	
Cl2	−0.03256 (6)	0.24404 (3)	0.07032 (6)	0.03440 (12)	
N2	0.5053 (2)	0.24052 (10)	0.5958 (2)	0.0307 (4)	
H10	0.5454	0.2843	0.6342	0.037*	
H8	0.5429	0.1967	0.6361	0.037*	
O1	0.26762 (16)	0.47238 (8)	0.57766 (15)	0.0273 (3)	
N1	0.32520 (17)	0.30954 (9)	0.40958 (17)	0.0219 (3)	
O3	0.60795 (16)	0.41098 (8)	0.36793 (17)	0.0288 (3)	
C7	0.1925 (2)	0.16842 (10)	0.2815 (2)	0.0247 (3)	
H7	0.1496	0.1214	0.2379	0.03*	
C3	0.3140 (2)	0.55353 (11)	0.26647 (19)	0.0232 (3)	
C1	0.2771 (2)	0.54062 (11)	0.63179 (19)	0.0225 (3)	
C8	0.1324 (2)	0.24062 (11)	0.2229 (2)	0.0235 (3)	
C9	0.2019 (2)	0.30880 (10)	0.2871 (2)	0.0234 (3)	
H9	0.1624	0.3565	0.2445	0.028*	
C5	0.3835 (2)	0.24049 (10)	0.4722 (2)	0.0226 (3)	
O4	0.57808 (18)	0.40136 (8)	0.65883 (16)	0.0313 (3)	
C6	0.3169 (2)	0.16920 (10)	0.4057 (2)	0.0234 (3)	
C4	0.1985 (3)	0.58896 (14)	0.1340 (2)	0.0343 (4)	
H5	0.2616	0.6087	0.0672	0.052*	
H6	0.1377	0.6313	0.165	0.052*	
H4	0.1226	0.5495	0.0855	0.052*	
O2	0.29538 (16)	0.48257 (8)	0.29448 (14)	0.0268 (3)	
C2	0.1372 (2)	0.56626 (12)	0.7009 (2)	0.0311 (4)	
H2	0.1617	0.6172	0.7448	0.047*	
H1	0.1249	0.5291	0.7752	0.047*	
H3	0.0361	0.5688	0.6264	0.047*	

### Atomic displacement parameters (Å^2^)

**Table:**

	*U*^11^	*U*^22^	*U*^33^	*U*^12^	*U*^13^	*U*^23^
Cu1	0.02168 (12)	0.01600 (12)	0.02151 (13)	0.00133 (6)	0.00540 (8)	0.00034 (7)
Cl1	0.0456 (3)	0.0184 (2)	0.0364 (3)	0.00489 (17)	0.0057 (2)	0.00646 (17)
Cl2	0.0360 (2)	0.0319 (3)	0.0301 (2)	0.00245 (18)	−0.00326 (19)	−0.00169 (18)
N2	0.0313 (8)	0.0209 (8)	0.0354 (9)	0.0017 (6)	−0.0016 (7)	0.0037 (6)
O1	0.0303 (6)	0.0243 (6)	0.0310 (7)	−0.0009 (5)	0.0144 (5)	−0.0015 (5)
N1	0.0219 (6)	0.0182 (7)	0.0267 (7)	0.0017 (5)	0.0077 (5)	0.0000 (6)
O3	0.0269 (6)	0.0249 (6)	0.0377 (7)	0.0009 (5)	0.0139 (5)	−0.0034 (6)
C7	0.0291 (8)	0.0191 (8)	0.0276 (9)	−0.0010 (6)	0.0101 (7)	−0.0033 (7)
C3	0.0244 (8)	0.0254 (8)	0.0203 (8)	0.0064 (6)	0.0059 (6)	0.0020 (6)
C1	0.0238 (8)	0.0237 (8)	0.0206 (7)	0.0049 (6)	0.0063 (6)	0.0043 (6)
C8	0.0236 (8)	0.0238 (8)	0.0234 (8)	0.0025 (6)	0.0060 (6)	0.0001 (6)
C9	0.0233 (7)	0.0203 (8)	0.0277 (8)	0.0034 (6)	0.0084 (6)	0.0016 (7)
C5	0.0226 (7)	0.0202 (8)	0.0267 (9)	0.0023 (6)	0.0091 (6)	0.0012 (6)
O4	0.0382 (7)	0.0223 (7)	0.0289 (7)	0.0014 (5)	−0.0019 (5)	0.0038 (5)
C6	0.0285 (8)	0.0156 (7)	0.0283 (9)	0.0039 (6)	0.0112 (7)	0.0037 (6)
C4	0.0334 (10)	0.0389 (11)	0.0278 (9)	0.0106 (8)	0.0009 (7)	0.0072 (8)
O2	0.0295 (6)	0.0239 (6)	0.0250 (6)	0.0007 (5)	0.0023 (5)	0.0022 (5)
C2	0.0301 (9)	0.0328 (10)	0.0346 (10)	0.0050 (7)	0.0159 (8)	−0.0004 (8)

### Geometric parameters (Å, °)

**Table:**

Cu1—O1	1.9665 (14)	C7—H7	0.93
Cu1—O4	1.9688 (15)	C3—O2	1.252 (2)
Cu1—O2	1.9691 (14)	C3—O4^i^	1.262 (2)
Cu1—O3	1.9743 (14)	C3—C4	1.505 (2)
Cu1—N1	2.2449 (15)	C1—O3^i^	1.259 (2)
Cu1—Cu1^i^	2.6600 (6)	C1—C2	1.511 (2)
Cl1—C6	1.7342 (18)	C8—C9	1.371 (3)
Cl2—C8	1.735 (2)	C9—H9	0.93
N2—C5	1.349 (2)	C5—C6	1.416 (3)
N2—H10	0.86	O4—C3^i^	1.262 (2)
N2—H8	0.86	C4—H5	0.96
O1—C1	1.261 (2)	C4—H6	0.96
N1—C9	1.348 (2)	C4—H4	0.96
N1—C5	1.351 (2)	C2—H2	0.96
O3—C1^i^	1.259 (2)	C2—H1	0.96
C7—C6	1.365 (3)	C2—H3	0.96
C7—C8	1.390 (2)		
			
O1—Cu1—O4	90.18 (6)	O3^i^—C1—O1	125.55 (16)
O1—Cu1—O2	86.83 (6)	O3^i^—C1—C2	117.59 (17)
O4—Cu1—O2	167.37 (6)	O1—C1—C2	116.85 (16)
O1—Cu1—O3	167.68 (6)	C9—C8—C7	119.85 (17)
O4—Cu1—O3	89.71 (7)	C9—C8—Cl2	120.30 (14)
O2—Cu1—O3	90.60 (6)	C7—C8—Cl2	119.83 (14)
O1—Cu1—N1	101.65 (6)	N1—C9—C8	122.72 (16)
O4—Cu1—N1	97.18 (6)	N1—C9—H9	118.6
O2—Cu1—N1	95.43 (6)	C8—C9—H9	118.6
O3—Cu1—N1	90.58 (6)	N2—C5—N1	119.58 (16)
O1—Cu1—Cu1^i^	83.91 (4)	N2—C5—C6	121.08 (16)
O4—Cu1—Cu1^i^	82.81 (5)	N1—C5—C6	119.34 (16)
O2—Cu1—Cu1^i^	84.67 (4)	C3^i^—O4—Cu1	124.50 (12)
O3—Cu1—Cu1^i^	83.85 (4)	C7—C6—C5	121.61 (16)
N1—Cu1—Cu1^i^	174.44 (4)	C7—C6—Cl1	119.62 (14)
C5—N2—H10	120	C5—C6—Cl1	118.77 (14)
C5—N2—H8	120	C3—C4—H5	109.5
H10—N2—H8	120	C3—C4—H6	109.5
C1—O1—Cu1	123.41 (12)	H5—C4—H6	109.5
C9—N1—C5	119.05 (15)	C3—C4—H4	109.5
C9—N1—Cu1	113.20 (11)	H5—C4—H4	109.5
C5—N1—Cu1	127.40 (12)	H6—C4—H4	109.5
C1^i^—O3—Cu1	123.07 (12)	C3—O2—Cu1	122.58 (12)
C6—C7—C8	117.37 (16)	C1—C2—H2	109.5
C6—C7—H7	121.3	C1—C2—H1	109.5
C8—C7—H7	121.3	H2—C2—H1	109.5
O2—C3—O4^i^	125.23 (16)	C1—C2—H3	109.5
O2—C3—C4	118.13 (17)	H2—C2—H3	109.5
O4^i^—C3—C4	116.64 (17)	H1—C2—H3	109.5

Symmetry codes: (i) −*x*+1, −*y*+1, −*z*+1.
